# Single-Shot Convolution Neural Networks for Real-Time Fruit Detection Within the Tree

**DOI:** 10.3389/fpls.2019.00611

**Published:** 2019-05-21

**Authors:** Kushtrim Bresilla, Giulio Demetrio Perulli, Alexandra Boini, Brunella Morandi, Luca Corelli Grappadelli, Luigi Manfrini

**Affiliations:** Dipartimento di Scienze Agrarie, University of Bologna, Bologna, Italy

**Keywords:** computer vision, deep learning, fruit recognition, harvesting robot, precision agriculture

## Abstract

Image/video processing for fruit detection in the tree using hard-coded feature extraction algorithms has shown high accuracy on fruit detection during recent years. While accurate, these approaches even with high-end hardware are still computationally intensive and too slow for real-time systems. This paper details the use of deep convolution neural networks architecture based on single-stage detectors. Using deep-learning techniques eliminates the need for hard-code specific features for specific fruit shapes, color and/or other attributes. This architecture takes the input image and divides into AxA grid, where A is a configurable hyper-parameter that defines the fineness of the grid. To each grid cell an image detection and localization algorithm is applied. Each of those cells is responsible to predict bounding boxes and confidence score for fruit (apple and pear in the case of this study) detected in that cell. We want this confidence score to be high if a fruit exists in a cell, otherwise to be zero, if no fruit is in the cell. More than 100 images of apple and pear trees were taken. Each tree image with approximately 50 fruits, that at the end resulted on more than 5000 images of apple and pear fruits each. Labeling images for training consisted on manually specifying the bounding boxes for fruits, where (x, y) are the center coordinates of the box and (w, h) are width and height. This architecture showed an accuracy of more than 90% fruit detection. Based on correlation between number of visible fruits, detected fruits on one frame and the real number of fruits on one tree, a model was created to accommodate this error rate. Processing speed is higher than 20 FPS which is fast enough for any grasping/harvesting robotic arm or other real-time applications.

**HIGHLIGHTS:**

Using new convolutional deep learning techniques based on single-shot detectors to detect and count fruits (apple and pear) within the tree canopy.

## Introduction

Agriculture is a sector with very specific working conditions and constraints. This is not only due to the dependency on the weather conditions, but as well on the labor market. During times of highly intensive agricultural activities (eg., harvest), there are very pronounced peaks in workload which can only be predicted on a short-term basis due to the weather conditions and seasonality. According to Schrder (2014), the world’s agricultural workforce is expected to decline around 30% between 2017 and 2030. This expected decline will be driven by structural changes within the agri-food industry, but also because the opportunities for employment are expected to be better in other sectors. Rural areas are already facing difficulties in creating attractive jobs in general, pushing toward an ongoing migration toward urban centers. Those structural changes in agriculture are expected to continue with higher investments in technology. For example, investing in precision farming and digital agriculture are expected to significantly increase (Colbert et al., 2016). New technologies are set to impact the farm labor dynamic in many ways (Pierpaoli et al., 2013), but two developments stand out. One, the increasing use of data collection tools, such as sensors, and increasing sophistication of farm hardware and software is increasing demand for higher analytical and technical skill sets (Aubert et al., 2012; Mulla, 2013). And two, the advancement of automation and autonomy on farm will decrease the reliance on human resources for low-skill and labor-intensive work while increasing autonomous machinery and robotics presence (Stafford, 2007; Bechar and Vigneault, 2016).

Along with many other emerging concepts of precision agriculture, the agricultural robot has evolved as one of the most promising of all (Tao and Zhou, 2017). Agriculture is most of the time repetitive, repetitive work of seeding, weeding, feeding, pruning, picking and harvesting, sorting, and so on. Agricultural robots automate those slow, repetitive and dull tasks for farmers, allowing them to focus more on strategic matters, and improving overall production yields (Edan et al., 2009). One of the most popular robotic application in agriculture are the autonomous harvesting and picking robots. That’s because the speed and accuracy has increased significantly in recent years (Bechar and Vigneault, 2016; Tao and Zhou, 2017). While the robots in addition to harvesting and picking can check at the same time the maturity level and sort based on size (Edan et al., 2009). However, there are many challenges for an autonomous robotic system to complete that task. In principle, for the robot to be fully capable to perform harvesting and picking, it needs a sophisticated detection algorithm in order to overcome challenges as naturally occurring changes in illumination, shape, pose, color, and viewpoint (Barnea et al., 2016).

Over the years, different approaches and techniques have been developed to tackle fruit detection and localization (Jimeìnez et al., 1999; Song et al., 2014). All the techniques until recently relied on feature extraction, be that, color, shape, reflectance, etc., (Song et al., 2014; Rahnemoonfar and Sheppard, 2017). Besides the aforementioned techniques, a new one which recently are gaining momentum and higher accuracy are deep learning (DL) techniques (Sa et al., 2016; Rahnemoonfar and Sheppard, 2017).

Deep learning is a subfield of machine learning (ML). While both fall under the broad category of artificial intelligence (AI), DL is what powers the most human-like AI applications. A DL model is designed to continually analyze data with a logic structure similar to how a human would draw conclusions. To achieve this, DL uses a layered structure of algorithms called an artificial neural network (ANN). The design of an ANN is inspired by the biological neural network of the human brain (LeCun et al., 2015). DL makes it possible to automatically learn the proper features from the photos and exploit them efficiently to construct an accurate detector through supervised training. Usually, the local visual features are extracted by a so called convolutional neural network (CNN), and a successive classifier consists of a fully connected network (FCN). CNNs preserve the spatial relationship between pixels by learning internal feature representations using small squares of input data. Feature are learned and used across the whole image, allowing for the objects in the images to be shifted or translated in the scene and remain detectable by the network (Krizhevsky et al., 2012).

Many of the state-of-the-art object detector CNNs are divided into two main groups. In one side, are models that reach higher accuracy but are slower: the two-stage detectors such as Faster R-CNN (Region-based Convolutional Neural Networks), and/or Mask R-CNN, that use a Region Proposal Network to generate regions of interests in the first stage and send the region proposals down the pipeline for object classification and bounding-box regression. In other side are models that reach lower accuracy but are faster: the single-stage detectors, such as You Only Look Once (YOLO) and Single Shot MultiBox Detector (SSD), that treat object detection as a simple regression problem which takes an input image and learns the class probabilities and bounding box coordinates.

In this paper it is presented a CNN model for fast and accurate fruit detection based on YOLO model (Redmon et al., 2015). By using those DL techniques, the need for hard-code specific features like specific fruit shapes, color and/or other attributes was eliminated. The network consists of several convolution and pooling layers, tweaked and changed from the standard model. Those modifications made to the model, make it more accurate to detect objects of the same class on close proximity (eg., only apple fruits, or only pear fruits). Even though the model was trained only on apple images (training data), it shows high accuracy on other fruits with similar attributes (green apples and green pears).

### Background and Related Work

The positions of the fruits in the tree are widely distributed, highly depending on the tree size, form, and growth. Furthermore, in addition to their position, fruits vary in size, shape, and reflectance due to the natural variation that exists in nature. Currently, no growth models can predict where fruit will occur. The shape of the fruit, one of the most distinctive features, varies between species and even cultivars (e.g., apples, oranges, etc., are cylindrical, but the width/height ratio are not constant with other fruits like pears) (Bac et al., 2014). Reflectance (mostly color and near-infrared) of fruit is a visual cue often used to distinguish fruit from other plant parts and still it varies strongly (Tao and Zhou, 2017). Color and texture are the fundamental character of natural images and plays an important role in visual perception. Color is often a distinctive and indicative cue for the presence of fruit. Most fruits when ripe have a distinctive color: red (apples, strawberries, and peaches, etc...), orange (oranges, etc...), or yellow (pears, lemons, peaches, and bananas). This makes them stand out from the green foliage when they are ready to pick (Edan et al., 2009; Barnea et al., 2016). However, some fruits even after ripening are still green (apple cv Granny Smith even after ripening does not change color) making them indistinguishable from the foliage on the basis of color alone (Edan et al., 2009).

The earliest fruit detection systems date since 1968 (Jimeìnez et al., 1999). Using different methods and approaches based on photometric information (light reflectance difference from fruits and leaves in visible or infrared spectrum), these detectors were able to differentiate fruits from other part of the tree. According to the reviews devoted to fruit detection by Jimeìnez et al. (1999) and Kapach et al. (2012) there were many problems related to growth habit, that had to be considered. The unstructured and uncontrolled outdoor environment also presents many challenges for computer vision systems in agriculture.

Light conditions have a major influence on fruit detection: direct sunlight results in saturated spots without color information and in shadows that cause standard segmentation procedures to split the apple surfaces into several fragments. In order to decrease the non-uniform illumination (daytime lighting can be bright, strong, directional, and variable), (Payne et al., 2014) described a machine vision technique to detect fruit based on images acquired during night time using artificial light sources. They reported 78% fruit detection, 10% errors and suggesting that artificial lighting at night can provide consistent illumination without strong directional shadows.

In a different approach, Kelman and Linker (2014) and Linker and Kelman (2015) presented an algorithm for localizing spherical fruits that have a smooth surface, such as apples, using only shape analysis and in particular convexity. It is shown that in the images used for the study, more than 40% of the apple profiles were none-convex, more than 85% of apple edges had 15% or more non-convex profiles, and more than 45% of apple edges had 50% or more non-convex profiles. Overall, 94% of the apples were correctly detected and 14% of the detection corresponded to false positives. Despite high accuracy number, the model is very specific to apples and would not be extensible to other fruit crops with less spherical shapes. Kapach et al. (2012) explains color highlights and spherical attributes, which tend to appear more often on the smoother, more secular, and typically elliptical regions like fruits where the surface normal bisects the angle between illumination and viewing directions. While a method for estimating the number of apple fruits in the orchard using thermal camera was developed by Stajnko et al. (2004). Si et al. (2015) describes location of apples in trees using stereoscopic vision. The advantage of the active triangulation method is that the range data may be obtained without much computation and the speed is very high for any robotic harvesting application. Jiang et al. (2008) developed a binocular stereo vision tomato harvesting in greenhouse. In this method, a pair of stereo images was obtained by stereo cameras and transformed to gray-scale images. According to the gray correlation, corresponding points of the stereo images were searched, and a depth image was obtained by calculating distances between tomatoes and stereo cameras based on triangulation principle. A similar method was described by Barnea et al. (2016) using RGB and range data to analyse shape-related features of objects both in the image plane and 3D space. In another work Nguyen et al. (2014) developed a multi-phase algorithm to detect and localize apple fruits by combining an RGB-D camera and point cloud processing techniques. Tao and Zhou (2017) developed an automatic apple recognition system based on the fusion of color and 3D features.

Until recent years, traditional computer vision approaches have been extensively adopted in the agricultural field. In recent years, with the significant increase in computational power, in particular with special purpose processors optimized for matrix-like data processing and large amount of data calculations (eg., Graphical Processing Unit – GPU), a lot of DL, CNN models and methodologies specifically have achieved breakthroughs never achieved before (LeCun et al., 2015).

Sa et al. (2016) developed a model called DeepFruits, for fruit detection. Adopting a Faster R-CNN model, goal was to build an accurate, fast and reliable fruit detection system. The model after training was able to achieve 0.838 precision and recall in the detection of sweet pepper. In addition, they used a multi-modal fusion approach that combines the information from RGB and NIR images. The bottle-neck of the model is that in order to deploy on a real robot system, the processing performance required is a GPU of 8 GB or more.

It is well known that all DL models, to have high accuracy they need high number of data (Krizhevsky et al., 2012). In case of CNN, the more images of the object of interest, the better the classification/detection performance is. In a model called DeepCount, Rahnemoonfar and Sheppard (2017) developed a CNN architecture based on Inception-ResNet for counting fruits. In order to use less training data, (Rahnemoonfar and Sheppard, 2017) used a different approach. They used another model to generate synthetic images/data to feed the main model to train on. Those generated images were simply a brownish and greenish color background with red circles drawn above it to simulate the background and tomato plant with fruit on it. They used twenty-four thousand generated images to feed into the model. The model after was tested on real world images and showed an accuracy from 85 to 80%.

To understand better the amount of data needed for better fruit detection, Bargoti and Underwood (2017) used different data augmentation techniques and transfer learning from other fruits. It is shown that transferring weights between different fruits did not have significant performance gains, while data augmentation like flip and scale were found to improve performance resulting in equivalent performance with less than half the number of training images.

## Materials and Methods

### Convolution Neural Networks

Convolutional neural networks are a specialized type of ANNs used for image analysis. Since computers sees image as a matrix of numbers that represent each pixel, it is important that the relation between the pixels (values) remains even after the image is processed through the network. To save this spatial relation between pixels, convolution neural networks are used that have different mathematical operations stacked on top of each-other to create layers of the network.

In the first part of this section it has been briefly explained the mathematical operations that serve as building blocks for the updated model architecture.

#### Convolution

Convolution is a mathematical operation that takes two functions ($K_{i,j}^{\left(l\right)}$ and $Y_{j}^{\left(l-1\right)}$) to produce a third one ($Y_{i}^{\left(l\right)}$). It is the process of adding each pixel of the image to its local neighbors, weighted by the kernel/filter of *m* × *n* size. In each layer, there is a certain number of filters. The number of filters *p* that is applied in one stage is equivalent to the depth of the volume of output feature maps. Each filter detects a particular feature at every location on the input. The output $Y_{i}^{\left(l\right)}$ of layer *l* consists of *p*^(*l*)^ feature maps of size *m*^(*l*)^ × *n*^(*l*)^. Thus the *i*^th^ feature map is computed as:

$$Y_{i}^{\left(l\right)}=B_{i}^{\left(l\right)}+\sum_{i=0}^{p}K_{i,j}^{\left(l\right)}\times Y_{j}^{\left(l-1\right)}$$

where $B_{i}^{\left(l\right)}$ is bias matrix and $K_{i,j}^{\left(l\right)}$ is the kernel connecting the feature map *j* of previous layer (*l* - 1) with *i*^th^ feature map in current layer *l*.

#### Activation

It is a function that takes the feature map $Y_{j}^{\left(l-1\right)}$ generated by the convolution and creates the activation map as output $Y_{i}^{\left(l\right)}$. It serves as a gate, to let certain part of the map elements pass while others not. This is strictly element-wise operation:

$$Y_{i}^{\left(l\right)}=f\left(Y_{i}^{\left(l-1\right)}\right)$$

where *f* is the function that have been used as a multiplier.

In the case of our model used, the activation layer is a special implementation of activation functions of non-linearity and rectification called PReLU:

$$Y_{i}^{\left(l\right)}=max\left(\alpha \times Y_{i}^{\left(l-1\right)},Y_{i}^{\left(l-1\right)}\right)$$

so, when $Y_{j}^{\left(l\right)}$ < 0, it will have a small positive slope of α.

#### Pooling

Pooling layer is used to reduce the computational requirements through the network and minimize overlapping by reducing the spatial size of the activation map. Pooling has two key components: spatial grouping *F*^(*l*)^ and spatial shift *S*^(*l*)^. It takes an input volume of *p*^(*l*-1)^ × *m*^(*l*-1)^ × *n*^(*l*-1)^ and provides a reduced volume output of size *p*^(*l*)^ × *m*^(*l*)^ × *n*^(*l*)^ where:

$$p^{\left(l\right)}=p^{\left(l-1\right)}\;\;\;\;\;\;\;n^{\left(l\right)}=\left(n^{\left(l-1\right)}-F^{\left(l\right)}\right)/S^{\left(l\right)}+1\;\;\;\;\;\;m^{\left(l\right)}=\left(m^{\left(l-1\right)}-F^{\left(l\right)}\right)/S^{\left(l\right)}+1$$

Most of the classical techniques for object detection used different filters/kernels to extract features and then programmatically apply detection. Those kernels, usually of 3 × 3 size, were manually given to the program to perform convolution with the image fed to it and then detect the objects of interests. Depending on the kernel type, those would extract features like edges, sharpening, color filtering, and many others (Lee and Rhodes, 1990). CNNs use techniques of loss function optimization and back propagation to automatically generate those kernels (in CNN called weights of the model). During this process the weights are updated with each iteration (epochs), until the best possible version is reached.

### Model Architecture

#### YOLO

**FIGURE 1:**
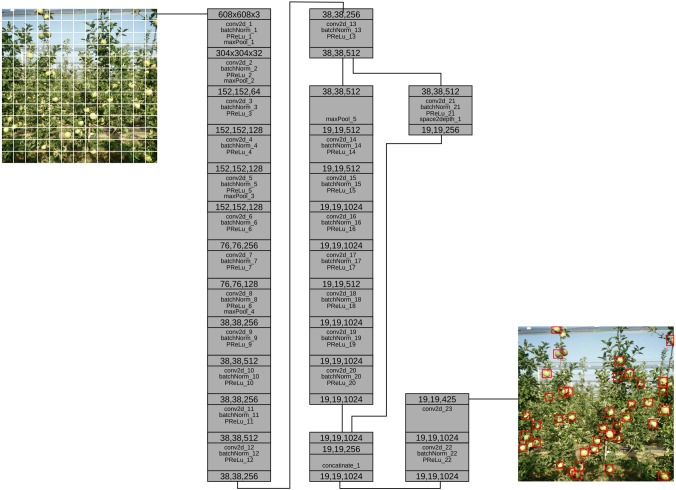
YOLO model with 24 Layers.

YOLO is a state-of-the art convolutional network for detection and localization. There are different versions of YOLO, and in this study we modified and used YOLO900 (also known as YOLOv2), and as such, in the remaining part of the paper, we refer to YOLO900 as YOLO. Compared to other state-of-the art methods that treat detection, classification and region extraction as different problems, YOLO does all in one pass (hence the name You Only Look Once) (Figure 1). To achieve that, YOLO in one hand loses in accuracy but on the other hand gains speed. YOLO takes as input an image of max size 608 × 608 and divides into *S* × *S*. Each grid cell is responsible for the bounding box whose center is at the location of the grid cell and predicts B bounding boxes as well as confidence level and class probability. In a dataset with *C* class labels, the output tensor is *S* × *S* × (*C* + *B* × 5). In our modified model, the class *C* is equal to 1 since we train the network each time with one class (just apples, just pears, etc.) which will be further discussed in the section below.

If the cell is offset from the top left corner of image by (*x*_c_, *y*_c_) and the bounding box ground truth is of size (*g*_w_, *g*_y_), then the prediction goes as:

$$\begin{array}{c} box_{x}=\sigma \left(x_{i}\right)+x_{c}\\ box_{y}=\sigma \left(y_{i}\right)+y_{c}\\ box_{w}=g_{w}e_{i}^{w}\\ box_{h}=g_{h}e_{i}^{h} \end{array}$$

The model assigns five anchor boxes to each cell. For each anchor box we need confidence score, four coordinates and the class number. *x*_i_ and *y*_i_ are the location of the center of the anchor box, *w*_i_ and *h*_i_ are the width and height of the anchor box *C*_i_ confidence score of whether there is an object or not, and *p*_i_(*c*) is the classification loss.

#### Updated Model

Despite being one of the most popular state-of-the art model, YOLO has problems detecting small objects (Redmon et al., 2015). The main problem with YOLO is that, the model can detect only one object class per cell, making it very difficult to detect two apples at the same cell. And since in apple tree, we are dealing with small fruits in relation to the canopy, we made different changes to the initial model, resulting in different accuracy scores.

The first methodology update (noted as model M1) was implemented to scale-up the grid. The standard YOLO takes input image and divides into 13 × 13. In our case we scaled up to 26 × 26. This improved the detection, as at this division of input image, the grid cell size is approximately similar in size to the apple fruit size. However, this almost doubled the training time, and decreased the overall detection speed. More so, the input image is still of size 608 × 608, it is just divided in finer grids. The second methodological update (noted as model M2) we took the M1 model and removed some layers, making the model shallower. We saw that, removing pooling layers and some other convolutional layers, we increase in speed while not losing in accuracy. In M2, we tried to make the model run on higher frames per second (FPS), in embedded devices (i.e., NVIDIA Jetson TX2). This network consists of the grid 26 × 26, while the network shallowness is reduced by half, instead of having 23 layers, in this model we have 11 layers. We removed pooling layers and used instead higher strides and kernel/filter size in convolution layers.

**FIGURE 2:**
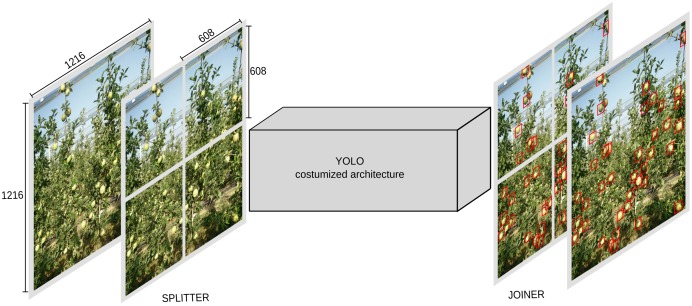
Model M3, with splitter and joiner blocks.

Moreover, (noted as model M3) we used M2 model as base, and then we added two other blocks (one in entrance and one in the end) (Figure 2). The entrance block noted as “splitter” and the end one noted as “joiner.” Splitter takes the image and separates it into four individual images. The resolution before split is 1216 × 1216 and then it gets splits into exactly four 608 × 608 images. The joiner at the end is responsible to put the four pieces together and output the results in 1216 × 1216 single image with detection. This technique of adding blocks to split and join the image introduced by Chen et al. (2018) is very effective and accurate, however, it decreases the speed of the network, as this in essence equals feeding four images instead of one.

### Dataset

Images were collected in the experimental orchards of University of Bologna – Department of Agricultural and Food Sciences (Italy), and in a commercial orchard in Ferrara (Italy). Multiple images of same tree have been taken with different angles, multiple fruit tree species and cultivars, multiple sources (webcam, DSLR camera, smartphone) and during different time of the year with different weather and light conditions (Table 1). In total 100 images of apples and 50 of pears were taken. Each image containing approximately 50 fruits, resulting in more than 5000 images to train the models. Images were taken before the coloring of apples occurred, thus all apples were still green. For pears, the number is comparably lower than apples, but in this case, we wanted to observe some transfer learning techniques from apples, thus this amount was enough to carry the study (Table 2).

**Table 1:** Image number, sources, and augmentation of apple fruits.

Camera	Number	Size	Resize	Augmented	Synthetic
Webcam	20	1280 × 720	608 × 608	80	0
Smartphone	100 + 30	2340 × 4608	1216 × 1216	200	100
DSLR	30	5664 × 8512	1216 × 1216	120	0


**Table 2:** Image number, sources, and augmentation of pear fruits.

Camera	Number	Size	Resize	Augmented	Synthetic
Smartphone	50	2340 × 4608	1216 × 1216	60	0


### Data Preparation

Data preparation and pre-analysis is the most important step in building ML algorithms. After images were collected together, we started the exploratory data analysis. Each image was shot in that way that it captures all the tree. However, 1/5 of image from top, and 1/5 from the bottom does not contain any apple fruits. In some cases, the bottom part even had fallen apples, that the model will try to detect, and later on results, would be considered as false positive and decrease the *F*_1_ score. To avoid that, and better fit the image in a square dimension all the images were cropped. In addition to cropping, the images had to be further resized to be suitable for the model. Depending on the model we used, the images were resized into two different sizes: 608 × 608 to be used by model M1 and M2 and 1216 × 1216 to be used by model M3. Everything was done with a script written in Python, that would take a batch of images and output the desired scaling and cropped shape.

Images are divided into Training set and Testing set. For Testing we used only images from smartphone camera. Those images went through all procedures of other images, until training the model. Those images are never shown to the training, as it is important for testing the accuracy of the models into images not seen before. This way we can see if the model is over-fitting on those training images or is still able to generalize and detect fruits on other images.

For labeling, we used a free and open-source labeling tool called BBox-Label-Tool where each image went through the process of labeling. Most problems during labeling are due to occlusion and overcrowded images. This happens when one object is either partially or completely occluded by the other, and when a large number of objects are close or attached to each other. Due to the nature of fruit trees, this is present on every image taken of that tree. In each image, every apple fruit visible was labeled with a bounding box representing the location of apple fruit. This is done manually and very carefully to avoid mislabeling or occlusion. However, this tool annotates the data into PASCAL Visual Object Classes (VOC) format, and in this study we adopted the DARKNET format.

#### Data Preparation

**FIGURE 3:**
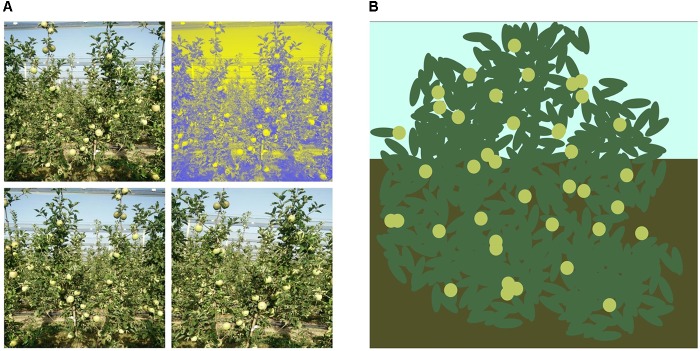
Input image augmentation. **(A)** Augmenting from an image to four. **(B)** Synthetic generated image.

Fetching the right amount and type of data which matches the use-case of our research is a difficult task. In addition, the data should have good diversity as the object of interest needs to be present in varying sizes, lighting conditions and poses for our network to generalize well during the training phase. To overcome this problem of limited quantity and limited diversity of data, we generated our own data with the existing data which we have. This methodology of generating our own data is known as data augmentation. There are numerous approaches to augment training data, in terms of quantity, it can be either by expanding the dataset with copies of augmented versions, or by randomly augmenting some data from the dataset. In terms of diversity, there are techniques like color manipulation, light and contrast, resizing, scaling, flipping, rotation, perspective transformation, adding noise, and so on. In this paper, we augmented all images in the dataset by randomly choosing the augmentation technique. For each image, three of those random augmentation was generated. In total 400 new images were created through augmentation (Figure 3A).

#### Data Set

As described by Rahnemoonfar and Sheppard (2017), deep-learning models require a lot of images to be collected and annotated then fed to the network. This is a very time-consuming and difficult task. In order for us to use less real images, we generated synthetic images for the network to train on. This is done automatically, through a Python script, where an image canvas of 608 × 608 was used. The upper part of background was colored with bluish color, representing the sky, while the lower part was colored with mixture of brown. Above that image, random elliptic dark-green shapes were generated representing leaves while random light-green and light-red circles were generated to represent fruits. In total 100 synthetic images were generated to observe if those data improve the detection of the models (Figure 3B).

### Training

YOLO originally is designed to run in DARKNET, which is an open-source DL library written in C. However, in our case we use YOLO in Keras with TensorFlow backend (another DL framework written in C and CUDA with Python bindings). Keras is a very high-level abstraction for many deep-learning frameworks that makes very easy for us to make changes in the network and test the changes immediately. In addition, with Keras we can easy use preprocessing techniques like transformation and augmentation before the image hits the first layer.

We trained the model on Amazon E3 cloud instance with NVIDIA Tesla K80 12 GB GPU. We used the stochastic gradient descent optimization method with 60 steps and the Adam optimizer with 0.002 learning rate to minimize the cost function. Choosing number of epoch is very difficult, as the number of epochs is related to the number of rounds of optimization that are applied during training. With more rounds of optimization, the error on training data will be reduced further; however, there is a point where the network becomes over-fit to the training images and will start to lose performance of generalization to unseen images. To avoid this, we monitored the error performance on testing images while the number of epochs increased. We ended up with 35 epochs as this resulted in the best accuracy while still maintaining the generalization on other images. No prior weights were used for training apple models, all weights were initialized randomly. While for pears, weights of apples were used.

#### Transfer Learning

Training CNNs is a difficult task both in terms of compute time required as well as computational resources required, especially on networks with a high number of layers. To avoid training the entire network again on different fruit (pears), we used two transfer-learning techniques called “fixed feature extractors” and “fine-tuning.”

Fixed feature extractors are the easier one, as it takes the trained network which shares the same architecture and uses it to classify or detect different class/objects that have never been trained on before. In this case we used weight learned and trained on apple fruits to detect pears.

Fine tuning takes the trained network weights which share the same architecture and transfers the weights directly to the new network we want to train with new data. We used the weights of network trained on apple as base of the network to train for the detection of pears (which has less amount of training data).

## Metrics and Results

The results will be evaluated using the data (images with labels) from the testing and validation set. The metrics evaluating the accuracy will be according to the well-known criteria based on Pascal VOC that much of the research on this field uses.

### Metrics

Pixel-wise accuracy was measured by comparison of ground truth and predicted information. Two metrics of accuracy are used: Confusion Matrix [precision, recall and *F*_1_ score, (Table 3)] and Intersect over Union (IoU). While for measuring speed, FPS is used.

**Table 3:** Confusion matrix.

		Predicted	
	
		True	False
GROUND TRUTH	TRUE	True Positive (TP)	False Negative (FN)
	FALSE	False Positive (FP)	True Negative (TN)


Precision evaluates the fraction of true positives (TP) detected bounding boxes in the pool of all true positives predictions TP and false positive predictions (FP) while recall evaluates the fraction of TP detected bounding boxes in the pool of all TP and false negatives predictions (FN):

$$precision=\frac{TP}{TP+FP}\;\;recall=\frac{TP}{TP+FN}$$

Precision and recall are tightly related; thus we can use only the *F*_1_ score, which takes in consideration both precision and recall to compute the score and how well the prediction fits the ground truth:

$$F_{1}=\frac{2\times precision\times recall}{precision+recall}$$

However, in order to compute the correctness of detection, we use IoU. IoU is defined by calculating the overlapping area of prediction and ground truth:

$$IoU=\frac{\left(p_{w}\times p_{h}\right)\cap \left(g_{w}\times g_{h}\right)}{\left(p_{w}\times p_{h}\right)\cup \left(g_{w}\times g_{h}\right)}$$

where *p*_w_ and *p*_h_ is the prediction bounding box width and height, and *g*_w_ and *g*_h_ is the ground truth bounding box width and height. A threshold above 0.5 IoU is considered as positive, while under is considered as poor detection. Another metric we used to measure speed is FPS. In this case, we tested how fast the model runs in a NVIDIA Jetson TX2 with 300 CUDA cores and NVIDIA GeForce 960M with 960 CUDA cores. FPS were calculated by dividing a second with the time in millisecond of processing a single image.

### Results

**FIGURE 4:**
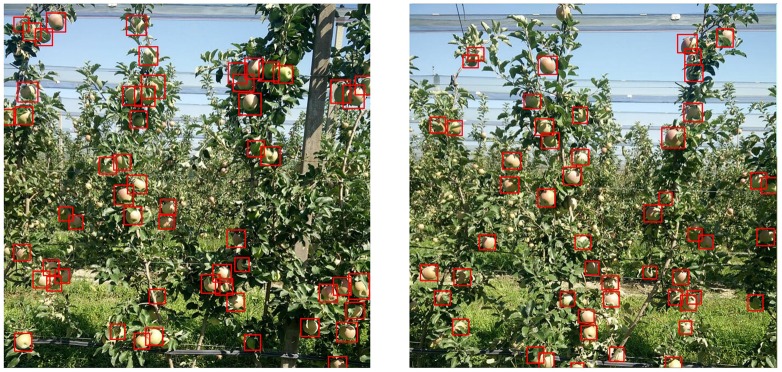
Apples detected from the updated model.

In this section we present the results obtained by comparing the different models used, their detection speed after training and the number of images the models are trained and transfer learning techniques (Figure 4).

#### Model Accuracy

**FIGURE 5:**
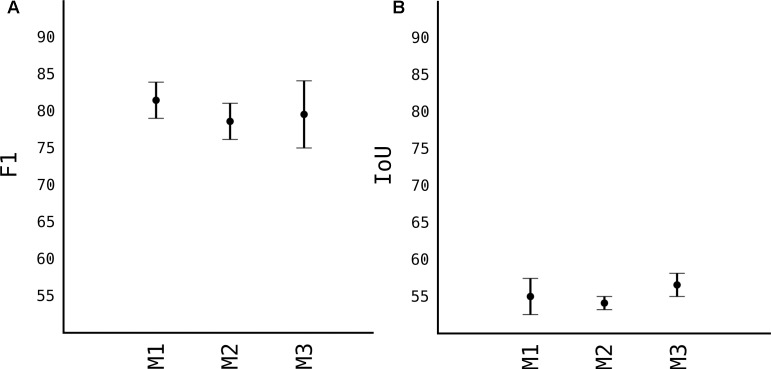
Comparison of accuracy of different models. **(A)** F1 accuracy score. **(B)** IoU accuracy score.

**FIGURE 6:**
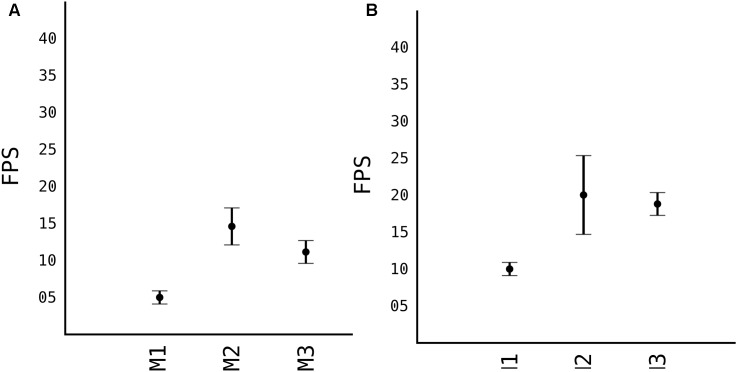
Running speed of models in different platforms. **(A)** Nvidia Jetson TX2. **(B)** Nvidia GeForce 960M.

Calculating accuracy and the speed of models, before choosing one to proceed, we compared all three models which were trained on 100 images of apples taken from sources mentioned in Table 1. As previously explained, stock YOLOv2 is not very accurate in detecting small objects with its standard 13 × 13 grid. However, first model M1 that uses 26 × 26 grid cell, is very accurate at detecting objects. As shown in Figure 5A, the *F*_1_ score of the M1 is 0.81 while having a relative high IoU score, as shown in Figure 5B. IoU in general tends to penalize single instances of bad classification more than the *F*_1_ score quantitatively even when they both are referring to the same bad detection instance. Since the image is resized 608 × 608 pixels, more pixers are erroneously classified as fruits, thus IoU penalizes them more than *F*_1_ score. With the grid 26 × 26 of model M1, speed suffers quiet a lot, see Figure 6. Average speed on Nvidia Jetson TX2 was 5FPS and on Nvidia GeForce 960M was 8FPS, which is very slow for any real time application use.

When we moved to M2, where we removed some layers from the model, in order to make it shallower, the model lost in *F*_1_ and IoU score. Respectively, the *F*_1_ is 0.77 while IoU is 0.53. And since the model is half less deep than M1, the processing speed from Figure 6, is significantly higher, with average FPS of 15 in TX2 and 20 in 960M.

When the “splitter” and “joiner” blocks are introduced to the M3 model, they improve on accuracy of M2, both in terms of *F*_1_ score and IoU score. *F*_1_ being 0.79 and IoU 0.58. However, we lose some FPS. This is due fact, that every image fed to the network, essentially is a 4-image split. Input size of the image is 1216 × 1216, and each of those 4 images of 608 × 608 pixels goes through network. This makes the model almost as accurate as model M1 while increasing the speed comparably from 8 FPS to 20 FPS (in Nvidia GeForce 960M).

**FIGURE 7:**
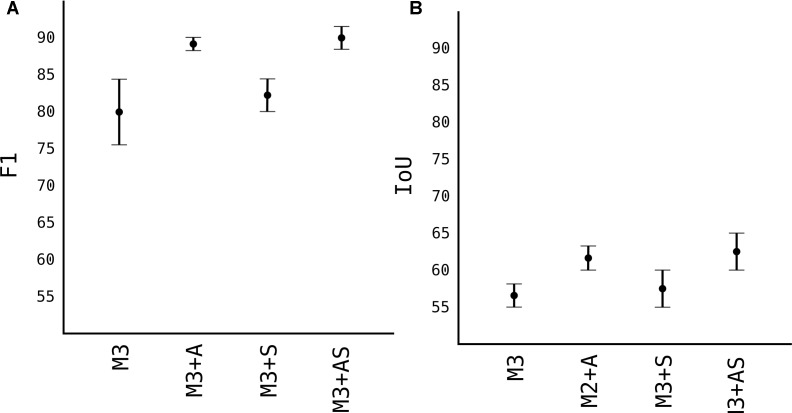
Image augmentation and synthetic images results. **(A)** F1 accuracy score. **(B)** IoU accuracy score.

For next results, we choose the M3 model to work with, and used other techniques to improve upon it, as showed in Figure 7.

#### Number of Images

Number of data is one of the most important factors in any DL model. Since the models generalize and learn patterns from labeled images, the quantity and quality of those data are of outmost importance. In the Figure 5, we used 100 images with approximately 50 fruits each, reaching *F*_1_ score of 0.79, which not very high. In Figure 7 we increased the number of images by augmentation and synthetic generated images and observed the model’s score. As expected, the *F*_1_ score of model M3 with 400 more augmented images (noted as M3+A) jumped from 0.79 to 0.89 and IoU from 0.57 to 0.62. When we added 100 synthetic generated images to the model M3 (noted as M3+S), the *F*_1_ score improved very slightly from 0.79 to 0.81, while IoU improved from 0.57 to 0.60. This shows that synthetic generated images helped the model better localize the pixels of the detected apple. When both 400 images from augmented part and 100 synthetic generated were added to M3 (together noted as M3+AS) the model showed the highest score observed, with *F*_1_ of 0.9 and 0.64 IoU.

#### Transfer Learning

**FIGURE 8:**
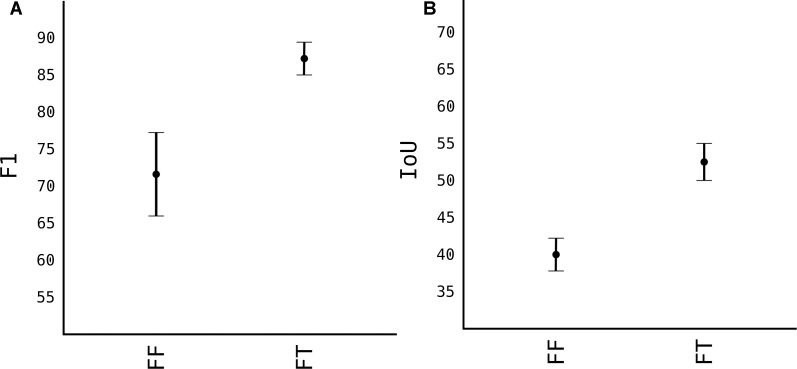
Transfer learning from M3+AS to pear images. **(A)** F1 accuracy score. **(B)** IoU accuracy score.

**FIGURE 9:**
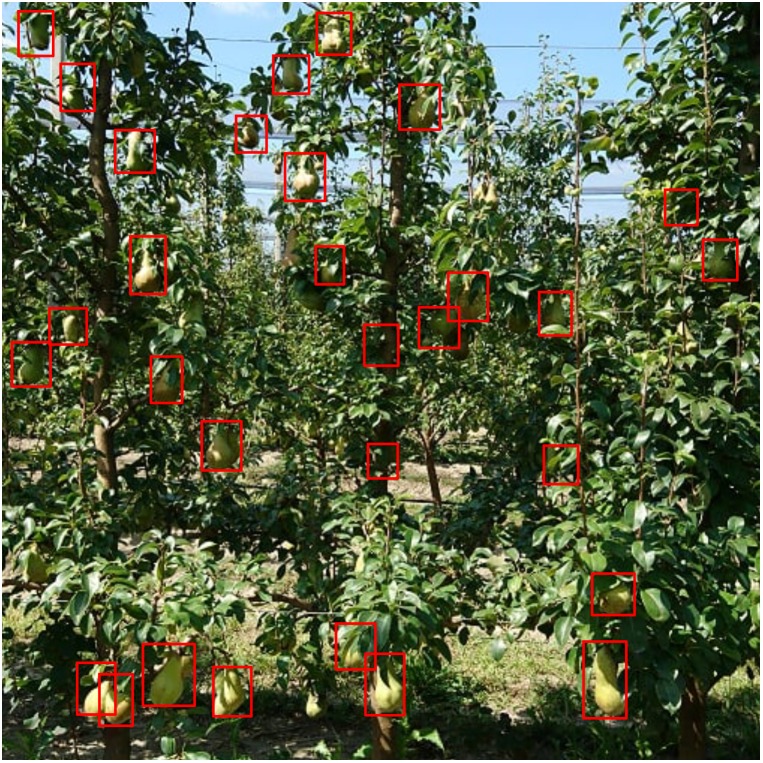
Pears detected from the updated model.

For pears, we only had 50 images. From which 40 of them were used for training, and we added another 60 from augmentation, resulting in around 5000 fruits, 15000 less than apples. This number of images, as shown in Figure 5 yield only an *F*_1_ of 0.8. To overcome that, we used transfer-learning techniques. Firstly, we used the weights of model M3+AS and tested in pears without any further training. Noted as FF (Fixed Feature extractor) from Figure 8, the results were surprisingly high with *F*_1_ score of 0.74. This showed that model M3+AS generalizes very well even in different fruit tree species even though never trained on images of that species (Figure 9). When the weights were further trained with very few additional images of pears, with just 5 more epochs the model reached an *F*_1_ score of 0.87 and IoU of 0.54.

### Cropload

Estimating fruit number based just on detected fruit from the model is not very accurate, despite the model being more than 90% accurate. This is not due to the model detection pipeline or architecture, but because of the nature of the tree itself, a problem known as fruit occlusion. The model is very accurate at detecting fruit even partially occluded, as the training data take into consideration even semi-occluded fruits, however, the problem remains with fully occluded fruits. When an image is taken of the tree, there are still apples that are not visible even by human looking at the image. Even looking at the tree from two meters far (the same distance as images were taken) it is difficult to see fruits inside the canopy. To solve this, we correlated the number of visible apples and hidden ones. We counted every fruit in a tree outside in the orchard where the images were taken, and then we counted again each fruit in the image (manually). We found out that, depending on the training system this number varies from 85% visible to 95% visible. Thus, we calculated tree cropload as below:

$$cropload=d_{i}+\left(d_{i}\times \left(1-F_{1}\right)\right)+\left(t_{i}\times \left(d_{i}+\left(d_{i}\times \left(1-F_{1}\right)\right)\right)\right)$$

where *d*_i_ is detection number by the model, *F*_1_ accuracy score, and *t*_i_ is percentage of hidden in that training system, in our case was 0.05 in one type of training system and 0.1 in another one.

## Conclusion

In this paper we presented an approach for fruit detection based on state-of-the art deep neural networks techniques using single shot detectors (YOLO) as a CNN to detect fruits of apple and pears in the tree canopy. This study demonstrates that modifications like the input grid on the standard model of YOLO yield better results. Furthermore, removing some layers of the model, we lose in accuracy but we gain in processing speed, this due to less compute resources needed to drive the model. In order to accommodate both speed and accuracy, we created another model, based on YOLO, with just 11 layers, with double grid size and introduced two new blocks to it. Those two blocks are completely independent and can be used with any other convolution neural network model. By splitting the image into smaller pieces and feeding separately to the model, the images retain higher resolution and are clearer. In addition, the objects in each individual block are bigger and easier to detect from the model. However, due to the limitation of the model we use, despite the modifications, the model is unable to detect if two objects of the same category (in our case we have just one category: apple fruits) are in the same grid cell.

By increasing the number of images, we increase the *F*_1_ and IoU score of the model. With 5000 images of apple fruits, the accuracy of the detection was *F*_1_ 0.79. With techniques like augmenting, we increased this number by four, into 20000 apple fruits, thus the *F*_1_ score reaches 0.9. In case when we added synthetic images, the *F*_1_ score remained the same, however, the IoU improved slightly. This shows that synthetic images can be an easy approach to fast generate images to improve the localization of pixels of detected object. Transfer-learning proved to be a very interesting tool in the fruit detection pipeline. Using the model trained solely in apple fruits, and later testing unchanged in pears, we observed *F*_2_ of 0.72. Using weights from apple models and training few epochs further in very small amount of pear images the *F*_2_ accuracy reached 0.87.

As the model is very dependent on the training system and the tree shape, the model is able to detect from 85% of fruits to 95%, thus its necessary to use the cropload model we developed to accommodate this change.

Current limitation of our platform is the compute power needed for the system to run. Because most neural networks have many layers, especially CNNs, the most suitable to run the models are the CUDA/OpenCL capable devices. Our continuation of this work will include more images of different fruit species, at different growth stages, with different training systems, and using a mobile platform for capturing those images. The earlier and smaller the fruit are, the more difficult is to detect. Indeed, it is very important for different orchard management procedures, to know the most precise cropload in order to proceed with more precision for the specific task.

## Author’s Note

This manuscript is a stem of Proceedings of the 14th International Conference on Precision Agriculture June 24 – June 27, 2018, Montreal, Quebec, Canada with title “Using DL – Convolutional Neural Networks (CNNS) for Real-Time Fruit Detection in the Tree” authored by KB, GP, AB, BM, LG, and LM.

## Author Contributions

The manuscript was done by KB and reviwed by LM and LCG. KB, LCG, AB, GDP, BM, LM carried the laboratory experiments. LM and LCG supervised the experiments.

## Conflict of Interest Statement

The authors declare that the research was conducted in the absence of any commercial or financial relationships that could be construed as a potential conflict of interest.
